# Commentary: Targeting LDH enzymes with a stiripentol analog to treat epilepsy

**DOI:** 10.3389/fncel.2015.00264

**Published:** 2015-07-07

**Authors:** Chang-Hoon Cho

**Affiliations:** School of Biosystem and Biomedical Science, College of Health Science, Korea UniversitySeoul, South Korea

**Keywords:** epilepsy, lactate, astrocyte, lactate dehydrogenase, ketogenic diet, oxamate, mTOR, circadian

Meeting energy demands in neurons is critical for proper functions of nervous systems. In addition to glucose, lactate is used as a major energy source in the brain, and a significant amount of lactate is produced through aerobic glycolysis in astrocytes (Schurr et al., 1988; Gladden, 2004; Ivanov et al., 2011; Dienel, 2012). It is reversibly converted to and from pyruvate by lactate dehydrogenase (LDH), and it is transported from astrocytes to neurons via the astrocyte-neuron lactate shuttle (Pellerin and Magistretti, 2012). Lactate is released from astrocytes through monocarboxylate transporters (MCT1 and MCT4), and has been also reported to be released through ion channels yet to be identified (Korn et al., 2005; Sotelo-Hitschfeld et al., 2015). Released lactate is taken into neurons through MCT2 and converted to pyruvate (Bergersen, 2007). Lactate also induces expression of genes (e.g., *arc* and *c-fos*) involved in synaptic plasticity (Suzuki et al., 2011; Yang et al., 2014).

In epilepsy where abnormal network activities of hyperexcitable neurons are uncontrollably synchronized, abundant energy for these activities has to be supplemented (Bertram et al., 1998). Expectedly, although more studies need to be done, high rates of glucose metabolism and elevated activity of LDH have been shown in people with epilepsy (PWE) and in animal models of epilepsy (Dufour et al., 2003). Increased level of lactate has been reported in some epilepsy cases (Hill et al., 1999). Other glycolytic enzymes are identified as markers for intractable temporal lobe epilepsies (e.g., neuron-specific enolases) and their defects (e.g., malic enzyme 2 and pyruvate dehydrogenase) are also shown either causative or susceptible to certain types of epilepsy (Steinhoff et al., 1999; Greenberg et al., 2005; Prasad et al., 2011).

Recently, Tsuyoshi Inoue's group in Japan reported that the anti-epileptic effect of the ketogenic diet (KD) bypass glycolysis (especially LDH), but occur elaborately through K_ATP_ channels–mediated mechanisms (Ma et al., 2007; Sada et al., 2015). They have shown in electrophysiological recordings that switching glucose to ketone bodies [ß–hydroxybutyrate (ß-HB) or acetoacetate] in artificial cerebrospinal fluid hyperpolarized resting membrane potentials in excitatory neurons, and reduced the firing rate of action potentials in acute brain slice preparations. Replacing ß-HB either with glucose or lactate returned the initial level of resting membrane potentials and the firing rate. Electrophysiological data recorded when oxamate, an LDH inhibitor, was included in the recording pipette showed similar effects to the ß-HB's data. This suggests that bypassing glycolysis in astrocytes, to supply energy for neurons, reduces the neuronal excitability. Paired recordings of astrocytes and neurons demonstrated that astrocytes are the site of LDH inhibition. Next, in kainate and pilocarpine models of epilepsy in mice, direct injection in the hippocampus or intraperitoneal injection of oxamate reduced the number of paroxysmal discharges (epileptiform activity), although the effect was short-lived (losing its drastic effect in an hour). Oxamate suppressed pilocarpine-induced acute behavioral seizures (*status epilepticus*) and prolonged its latency. It also suppressed paroxysmal discharges in a chronic epilepsy model. The antisense oligodeoxynucelotide targeting LDHA, one of two major genes composed of LDH, was injected into the hippocampus of kainate model, suppressing spontaneous spikes. This confirms that LDH is a critical target. Next, they screened clinically available anti-epileptic drugs for inhibiting activities of purified LDH, and found that Stiripentol partially inhibits LDH. Isosafrole, a Stiripentol derivative that has a greater LDH inhibitory effect than Stiripentol does, suppressed spontaneous spikes dramatically in kainate model (Sada et al., 2015). This is a significant step forward elucidating the metabolic mechanism of epilepsy. Although LDH is not the first metabolic enzyme shown to be important in epilepsy, its inhibition was the first to suppress the epileptiform discharges *in vivo* (Novarino et al., 2012; Papetti et al., 2013).

In epilepsy, hypoxia-inducing factor 1α (HIF1α) has been induced in reactive astrocytes (Vangeison et al., 2008; Li et al., 2014). Several glycolytic enzymes, including LDH, have been shown to be upregulated by HIF-1α, although HIF-1α-mediated LDH induction needs to be verified in PWE and animal models of epilepsy (Marín-Hernández et al., 2009). Interestingly, oxamate inhibits not only LDH but also mTOR pathway, which is the major signaling pathway in both genetic and acquired epilepsies (Cho, 2011; Zhao et al., 2015). In addition, activation of mTOR pathway increases the expression of LDH by activating STAT3, a transcription factor and downstream target of mTOR pathway, which has been shown to be activated in epilepsy (Lund et al., 2008; Zha et al., 2011). Furthermore, rapamycin, an mTOR inhibitor, reduces lactate level by decreasing the activity and expression of LDH and/or inhibiting hexokinase II, an upstream enzyme of glycolysis (Venkatesh et al., 2012; Lee et al., 2013). Therefore, there are interactions between the mTOR pathway and lactate/LDH, which need to be explored further. Finally, metabolic enzymes in glycolysis are regulated in a circadian manner (Zhang et al., 2009). Particularly, LDH activity and its mRNA expression follow the circadian pattern in suprachiasmatic nucleus (Isobe et al., 2011). It is entertaining to imagine that if the level of lactate in the brain is higher at night than during the day, nocturnal types of epilepsy might be better explained (Isobe et al., 2011; Cho, 2012).

There are many follow-up questions that remain to be addressed. First, what would be the side-effect of prolonged inhibition of LDH for controlling epileptic seizures? Will cells in other organs (e.g., heart and muscle) function properly with LDH inhibitors? If not, how we can make this work only on (reactive) astrocytes in the epileptic foci? Second, will isosafrole have enhancing effects of GABAergic synaptic transmission like stiripentol (Quilichini et al., 2006; Fisher, 2009; Grosenbaugh and Mott, 2013)? Third, although lactate in the hippocampus has been shown to be decreased by KD without affecting the level and the activity of LDH in this study, it remains to be seen that the level of lactate is lowered by KD in PWE and animal models of epilepsy.

## Conflict of interest statement

The author declares that the research was conducted in the absence of any commercial or financial relationships that could be construed as a potential conflict of interest.
